# Metabolomics Analysis Reveals the Protection Mechanism of Huangqi–Danshen Decoction on Adenine-Induced Chronic Kidney Disease in Rats

**DOI:** 10.3389/fphar.2019.00992

**Published:** 2019-09-10

**Authors:** Xinhui Liu, Bing Zhang, Shiying Huang, Fochang Wang, Lin Zheng, Jiandong Lu, Youjia Zeng, Jianping Chen, Shunmin Li

**Affiliations:** ^1^Department of Nephrology, Shenzhen Traditional Chinese Medicine Hospital, Guangzhou University of Chinese Medicine, Shenzhen, China; ^2^The Fourth Clinical Medical College, Guangzhou University of Chinese Medicine, Shenzhen, China; ^3^Shenzhen Key Laboratory of Hospital Chinese Medicine Preparation, Shenzhen Traditional Chinese Medicine Hospital, Guangzhou University of Chinese Medicine, Shenzhen, China

**Keywords:** chronic kidney disease, traditional Chinese medicine, Huangqi–Danshen decoction, metabolomics, ultra-high-performance liquid chromatography, mass spectrometry

## Abstract

Huangqi–Danshen decoction (HDD) is a commonly used drug pair for clinical treatment of chronic kidney disease (CKD) in traditional Chinese medicine with good efficacy. However, the potential mechanisms of this action have not been well elucidated. The aim of this study was to explore the metabolic profiling variations in response to HDD treatment in a CKD rat model. CKD rat model was induced by adding 0.75% adenine to the diet for 4 weeks. The rats in the treatment group received HDD extract orally at the dose of 4.7 g/kg/day during the experiment. At the end of the experiment, serum and kidney samples were collected for biochemical and pathological examination. Ultra-high-performance liquid chromatography coupled with quadrupole time-of-flight mass spectrometry (UHPLC-QTOF/MS) was used to analyze metabolic profiling variations in the kidney. The results showed that treatment with HDD markedly attenuated kidney injury and improved renal function. A total of 28 metabolites contributing to CKD phenotype were found and identified in the kidney samples. The primary metabolic pathways disordered in the kidney of CKD rats were glycerophospholipid metabolism, glycosylphosphatidylinositol-anchor biosynthesis, and citrate cycle. Partial least squares discriminant analysis (PLS-DA) score plot showed that the three groups of renal samples were obviously divided into three categories, and the metabolic trajectory of the HDD treatment group moved to the control group. (E)-Piperolein A, phosphatidylcholines (PC) (18:1/22:6), phosphatidylinositols (PI) (13:0/18:1), PI (15:0/20:3), phosphatidylserines (PS) (O-20:0/12:0), and triglyceride (TG) (22:4/24:0/O-18:0) represented potential biomarkers of the renoprotective effects of HDD against CKD. In conclusion, HDD has renoprotective effect against adenine-induced CKD, which may be mediated *via* partially restoration of perturbed metabolism in the kidney.

## Introduction

Chronic kidney disease (CKD) arises from many heterogeneous diseases that alter the function and structure of the kidney irreversibly, over months or years (Webster et al., 2017). According to The 2017 Global Burden of Disease (GBD) Study, global absolute CKD prevalence increased by 28.2% from 2007 to 2017 among women and 25.4% among men (James et al., 2018), and the number of global deaths due to CKD will rise from 1.2 million in 2016 to 3.1 million in 2040 (Foreman et al., 2018). The high prevalence and mortality of CKD makes its treatment an urgent task. Normal strategies for the treatment of CKD have emerged over the past years, such as endothelin antagonists, vitamin D receptor agonists, anti-inflammatory agents, bardoxolone, pentoxifylline, etc. Despite promising results in experimental models and small randomized studies, adequately powered randomized trials are required to evaluate the benefits and risks of these therapies in the CKD population (Lambers Heerspink and De Zeeuw, 2013; Pena-Polanco and Fried, 2016). The use of traditional Chinese medicine (TCM) has been a major complementary and alternative branch of CKD therapy (Li and Wang, 2005; Zhong et al., 2013; Zhong et al., 2015). Increasing evidence indicates the beneficial effects of TCM on prevention and treatment of CKD in both clinical CKD patients (Wang et al., 2012; Hsieh et al., 2014; Lin et al., 2015; Zheng et al., 2017) and CKD animal models (Zhao et al., 2015a; Wang et al., 2017b; Dou et al., 2018; Liu et al., 2018; Zhang et al., 2018). Huangqi–Danshen decoction (HDD), a TCM drug pair, is composed of Astragali Radix (Huang-qi) and Salviae Miltiorrhizae Radix et Rhizoma (Dan-shen). According to TCM theory, HDD possesses the efficacies of replenishing Qi and activating blood (Yi-Qi-Huo-Xue), which helps delay the progress of CKD. The previous pharmacological study has showed the renoprotective effect of HDD in adenine-induced CKD rats (Liu et al., 2019). However, the underlying mechanism of HDD on CKD is less known.

Metabolomics has been used to characterize the biochemical metabolites related to pathogenesis of disease that could be used for the diagnosis and monitoring of the disease progression and response to therapeutic interventions (Johnson et al., 2016). In agreement with the holistic thinking of TCM, metabolomics has shown potential in bioactivity evaluation and action mechanism of TCM as well as pharmaceutical research and development (Wang et al., 2017a). Recently, ultra-high-performance liquid chromatography coupled with quadrupole time-of-flight mass spectrometry (UHPLC-QTOF/MS)-based metabolomics has been widely applied to evaluate therapeutic effects and potential mechanisms of TCM formulas or herbs in treating CKD (Zhang et al., 2015; Zhao et al., 2015b; Dou et al., 2018; Zhang et al., 2018). Therefore, metabolomics provides an analytical platform in understanding the mechanism of action of Chinese herbal medicine, which will help to promote the modernization of TCM.

In the present study, addition of 0.75% adenine to the diet of rats was used to cause metabolic abnormalities similar to human CKD (Chen et al., 2016). Blood biochemical parameters and kidney pathological changes were used to evaluate the characteristics of CKD and efficacy of HDD treatment. Finally, untargeted metabolomics approach was applied to investigate the metabolic profiling and the response to HDD treatment in adenine-induced CKD rat model.

## Materials and Methods

### Chemicals and Reagents

Adenine was purchased from Sigma-Aldrich (St. Louis, MO, USA). The primary antibodies used in the present study included fibronectin (FN), type IV collagen (Col-IV) (abcam, Cambridge, MA, USA), and α-smooth muscle actin (α-SMA) (Sigma-Aldrich, St Louis, MO, USA). HDD consists of Astragali Radix [roots of *Astragalus membranaceus* (Fisch). Bge. var. *mongholicus* (Bge). Hsiao] and Salviae Miltiorrhizae Radix et Rhizoma (roots and rhizomes of *Salvia miltiorrhiza* Bge). (**Table 1**). All the botanical names are recorded and can be validated using http://mpns.kew.org/mpns-portal/?_ga=1.111763972.1427522246.145907734. The HDD water extract was prepared as previously described (Liu et al., 2019). High-performance liquid chromatography–mass spectrometry (HPLC-MS) analysis was conducted to confirm the quality of the HDD extract, as indicated in **Supplementary Figure S1**.

**Table 1:** The herbal composition and proportion of HDD.

Botanical name	Herbal name	Chinese name	Voucher number	Dosage
*Astragalus membranaceus* (Fisch). Bge. var. mongholicus (Bge). Hsiao	Astragali Radix	Huang-Qi	2010015Z	30 g
*Salvia miltiorrhiza* Bge.	Salviae Miltiorrhizae Radix et Rhizoma	Dan-Shen	2010006Z	15 g

### Animals

All animal experiments were carried out in accordance with protocols approved by the Ethics Committee of Shenzhen Traditional Chinese Medicine Hospital, Guangzhou University of Chinese Medicine (Shenzhen, China), and all efforts were made to minimize animal suffering. Eight-week-old male Sprague–Dawley (SD) rats were randomly divided into the following three groups: control group (*n* = 6), CKD group (*n* = 6), and CKD + HDD group (*n* = 6). Rats in the CKD and CKD + HDD group were fed a diet containing 0.75% w/w adenine for 4 weeks. CKD + HDD group was administered with HDD extract (4.7 g/kg/day) by gastric irrigation during 4 weeks study period. Control group rats received normal adenine-free feed for 4 weeks. At the end of study, all rats were anesthetized (sodium pentobarbital, 75 mg/kg, intraperitoneal injection), and blood samples were obtained by cardiac puncture. The rats were euthanized by cervical dislocation without regaining consciousness. The kidneys were rapidly harvested and processed for histological examination and metabolomic analysis.

### Serum Biochemical Analysis

Serum samples were isolated from blood by centrifugation for 10 min at 1,000 rpm at 4°C. Serum creatinine (Scr), blood urea nitrogen (BUN), and albumin were measured by creatinine serum detection kit, BUN detection kit (StressMarq Biosciences, British Columbia, Canada), and QuantiChrom™ BCG Albumin Assay Kit (BioAssay Systems, Hayward, CA, USA), respectively, following the manufacturer’s instructions.

### Histological Analysis

Paraffin-embedded kidneys from rats of three groups were cut into 4-µm sections, dewaxed, and rehydrated. Sections were stained with periodic acid–Schiff (PAS) and Masson’s trichrome stains. The degree of tubular atrophy in PAS staining was determined based on a scale of 0–3 points: 0, normal tubules; 1, rare single atrophic tubule; 2, several clusters of atrophic tubules; and 3, massive atrophy (Lu et al., 2018). Interstitial fibrosis area in Masson staining was measured using Image J software (NIH, Bethesda, MD, USA). Five microscopic fields (200×) of each rat and six rats in each group were performed quantitative analyses in a blinded manner.

### Immunohistochemistry

The paraffin-embedded rat kidney slides were dewaxed, rehydrated, and immersed in 3% hydrogen peroxide for 10 min at room temperature to block endogenous peroxidase activity. Then, the slides were blocked with 10% goat serum for 1 h at 37°C and were stained with primary antibodies against fibronectin (FN) (dilution 1:200), type IV collagen (Col-IV) (dilution 1:200), and α-smooth muscle actin (α-SMA) (dilution 1:250) at 4°C overnight followed by SignalStain Boost Detection Reagent (Cell Signaling Technology, Beverly, MA, USA) for 30 min at room temperature. The sections were then treated with SignalStain diaminobenzidine (DAB) substrate (Cell Signaling Technology, Beverly, MA, USA) and counterstained with hematoxylin. The integrated optical density (IOD) values of the positive staining areas were measured by ImagePro Plus 6.0 software (Media Cybernetics, CA, USA). Five microscopic fields (200×) of each rat and three rats in each group were counted in a blinded manner.

### Sample Extraction for UHPLC-QTOF/MS Analysis

Renal tissue samples were ground by liquid nitrogen at room temperature, and then 20 mg of the renal tissue was added with 800 μl ice-cold mixture of methanol, acetonitrile, and acetone (1:1:1, v/v). The mixture was thoroughly vortexed for 2 min, incubated at 4°C for 10 min, then centrifuged (14,000 rpm for 10 min at 4°C). The supernatant (600 μl) was transferred and then evaporated to dryness under nitrogen gas at 37°C. The residue was reconstituted with 200 μl 10% acetonitrile and centrifuged (13,000 rpm for 10 min at 4°C) after being vortexed for 60 s. The supernatant was transferred to autosampler vials for further analysis.

### Sample Preparation and Extraction of Quality Control Sample

To guarantee the quality of the nontargeted bioanalytical data, quality control (QC) samples were used for method validation. Therefore, 20 mg mixture of renal tissue samples from each group was pooled to obtain a QC sample, and then, 110 μl of the QC sample was added with 10 μl IS (2,4-dichloro-phenylalanine, 0.2 mg/ml). QC samples were extracted using the sample extraction method mentioned above. The QC specimens were inserted between every six experimental samples throughout the whole analysis procedure.

### UHPLC-QTOF/MS Analysis

Chromatographic separation was performed on a Dionex UltiMate 3000 UHPLC system (Thermo Scientific, San Jose, CA, USA). UHPLC conditions were as follows: column: Waters Acquity BEH C18 column (2.1 × 100 mm, 1.7 μm, Milford, MA, USA); column temperature: 30°C; mobile phase: 0.1% formic acid water (A) and acetonitrile (B); gradient conditions for positive mode analysis: 0–4 min 10–13% B; 4–6 min 13–50% B; 6–10 min 50% B; 10–14 min 50–85% B; 14–18 min 85% B; 18–20 min 85–100%; 20–25 min 100%; re-equilibrate: 10 min; gradient conditions for negative mode analysis: 0–2 min 15% B; 2–4 min 15–55% B; 4–8 min 55% B; 8–14 min 55–70% B; 14–22 min 70–80% B; 22–24 min 80–100%; 24–28 min 100%; re-equilibrate: 10 min; flowrate: 0.2 ml/min; injection volume: 2 μl.

MS detection was performed on an Orbitrap mass spectrometer (Thermo Fisher Scientific, San Jose, CA, USA), which was operated in both positive (ESI^+^) and negative electrospray ionization interface (ESI^−^). The MS parameters were as follows: heater temperature, 350°C; sheath gas flowrate, 35 arb; aux gas flowrate, 10 arb; sweep gas flowrate, 0 arb; I spray voltage, 3.2 kV; capillary temperature: 300°C; mass range: m/z 150–1,500.

### Data Processing

The acquired raw data were preprocessed by the following procedures: peaks identification, peaks filtration and normalization to total area, and peaks alignment. The three-dimensional data set included sample information, peak intensities, peak retention time (RT), and mass-to-charge ratio (m/z). Then, the three-dimensional data matrices were imported into the SIMCA14.1 (Umetrics, Umeå, Sweden) software for multivariate statistical analysis. The analysis methods including principal components analysis (PCA), partial least-squares discriminant analysis (PLS-DA), and orthogonal partial least-squares discriminant analysis (OPLS-DA) were used for metabolic profile analysis.

### Biomarker Identification and Metabolic Pathway Analysis

OPLS-DA model was used to visualize the metabolic difference between the control group and the model group. Variables with VIP >1 in the OPLS-DA model, variables with p|corr| value >0.58 in S-plot, and variables with cross-validation by jack-knife method were considered as potential biomarkers. The potential biomarkers were identified using Masslynx (Waters, Milford, MA, USA) combined with the METLIN database^1^ and Human Metabolome Database (HMDB)^2^. Then, receiver operating characteristic (ROC) curves (Prism 7.00) were applied to analyze data for evaluating the predictive power of the identified biomarkers. Pathway analysis was based on the Kyoto Encyclopedia of Genes and Genomes (KEGG)^3^ and MetaboAnalyst^4^.

### Statistical Analysis

Unless otherwise stated, data were expressed as mean ± standard error of mean (SEM). The statistical analyses were performed using SPSS statistics software version 16.0 (SPSS Inc., Chicago, IL, USA). Comparison of the same parameter among groups was analyzed by one-way ANOVA followed by *post hoc* analysis with Tukey test. The value of *P* < 0.05 was considered statistically significant.

## Results

### Standardization of HDD Extract

Before drug treatment, HDD was first chemically standardized. An HPLC-MS approach was established to reveal the chemical profile of HDD extract and quantify the main ingredients in the extract. Using the respective individual standard, 11 compounds were identified from HDD extract (**Supplementary Figure S1**), and the minimum amount in mg/g of dried extract was 0.01659 for protocatechualdehyde, 0.0052 for caffeic acid, 0.0574 for rosmarinic acid, 0.0436 for lithospermic acid, 0.0779 for ononin, 0.0184 for calycosin 7-O-beta-D-glucoside, 0.4503 for salvianolic acid B, 0.0015 for calycosin, 0.0018 for astragaloside IV, 0.0017 for astragaloside III, and 0.0088 for astragaloside II. The chemical analysis of HDD extract here served as quality control for the reproducibility of the below animal study.

### HDD Attenuated Adenine-Induced CKD in Rats

**Figure 1:**
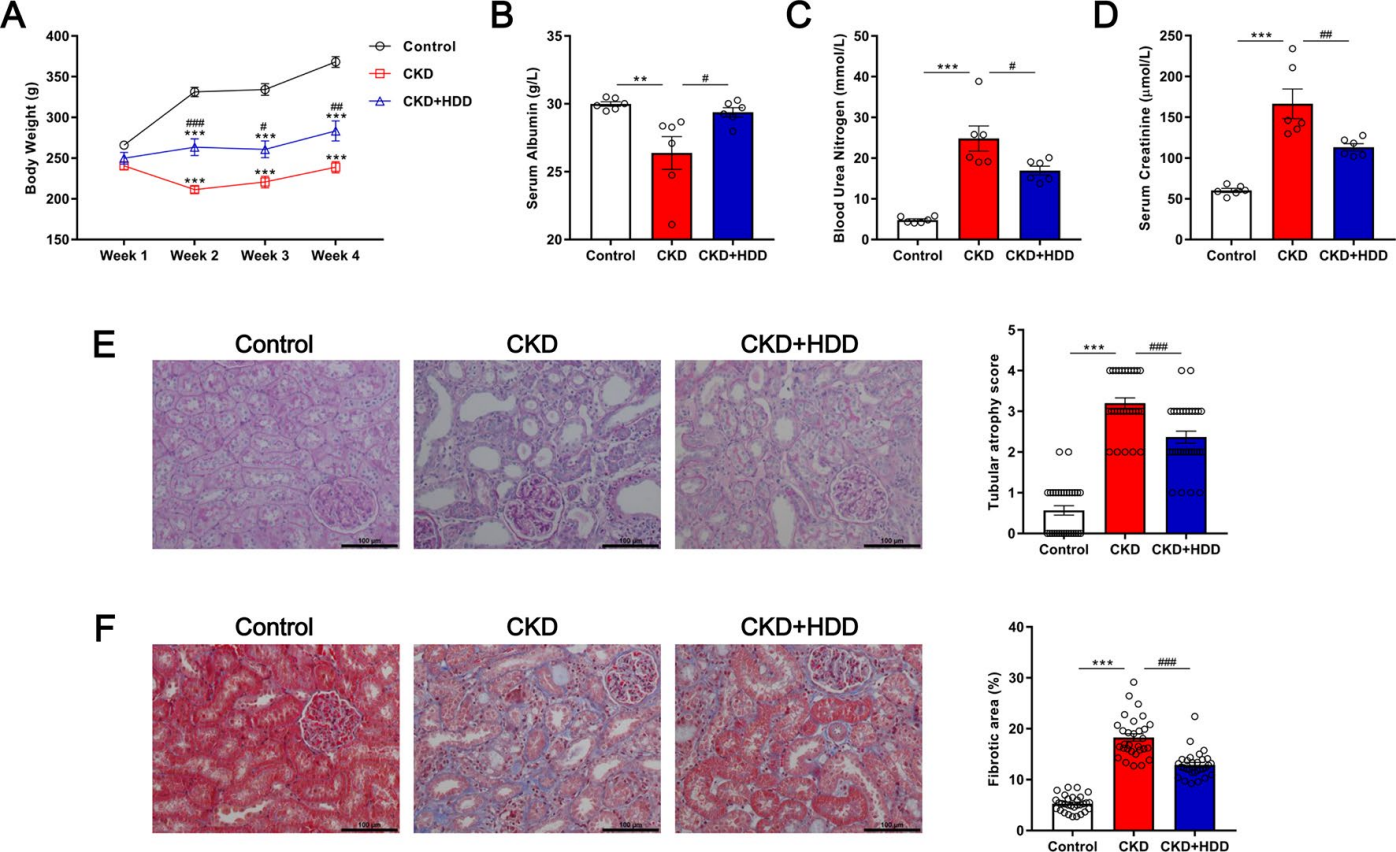
Effects of HDD on adenine-induced CKD. **(A)** Body weight. **(B)** Serum albumin. **(C)** Blood urea nitrogen. **(D)** Serum creatinine. **(E)** PAS staining. **(F)** Masson staining. Data are presented as the means ± SEM, n = 6 rats per group (**P < 0.01, ***P < 0.001 compared with the control group; ^#^*P* < 0.05, ^##^*P* < 0.01, ^###^*P* < 0.001 compared with the CKD group).

Adenine-induced CKD rats began to lose weight significantly from the second week until the end of the experiment. HDD treatment obviously prevented the decline of body weight in CKD rats (**Figure 1A**). Consistently, we observed that HDD increased serum albumin level, a nutritional indicator, by 11.37% in CKD rats (**Figure 1B**). BUN and Scr levels were measured to evaluate the renal function. As shown by the data, BUN level significantly increased by 5.16-fold in the CKD group compared with the control group and was blocked by 31.65% in the CKD + HDD group (**Figure 1C**). Similarly, Scr level increased by 2.76-fold in CKD rats and was blocked by 32.01% after HDD treatment (**Figure 1D**). In agreement with the improved renal function, massive tubular atrophy in PAS staining and obvious fibrosis in Masson staining in CKD rats were significantly reversed by HDD treatment (**Figures 1E, F**). Moreover, the potential side effects of HDD were estimated by the levels of aspartate transaminase (AST) and alanine transaminase (ALT). There were no significant differences in ALT and AST levels between the three groups (**Supplementary Figure S2**). These data demonstrated that HDD attenuated adenine-induced CKD in rats without toxic effect on liver function.

### HDD Inhibited Tubulointerstitial Fibrosis in CKD Rats

**Figure 2:**
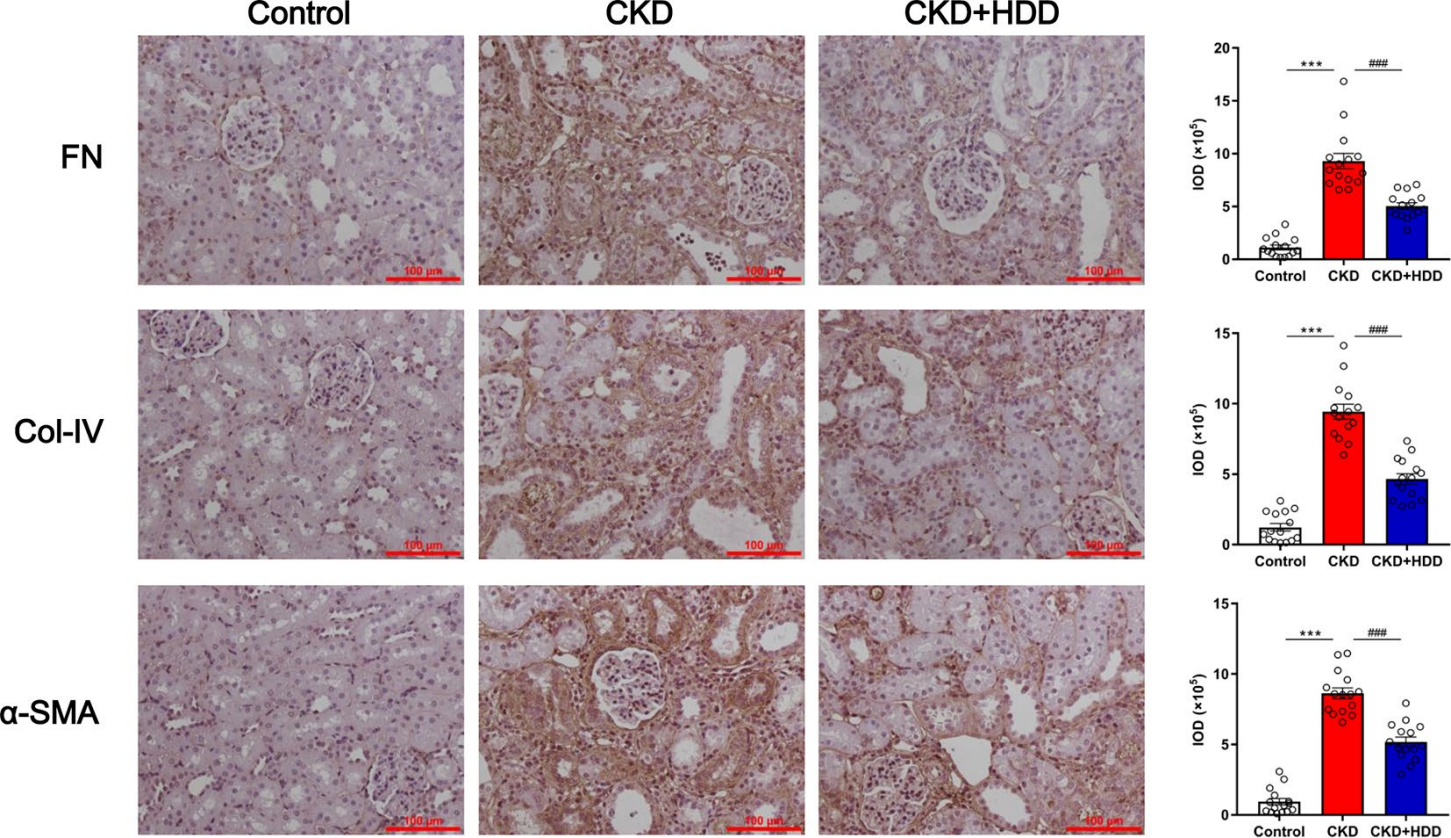
HDD inhibited fibrotic markers expression in CKD rats. Data are presented as the means ± SEM, n = 3 rats per group (***P < 0.001 compared with the control group; ^###^*P* < 0.001 compared with the CKD group).

Tubulointerstitial fibrosis (TIF) is the common final pathway for CKD progression to end-stage renal disease (ESRD). Immunohistochemistry staining showed that TIF markers FN, Col-IV, and α-SMA were significantly increased in the kidney of CKD rats and were strikingly inhibited by 45.77, 50.61, and 39.94%, respectively, after HDD administration (**Figure 2**). These data indicated that HDD inhibited TIF in CKD rats.

### Repeatability and Stability of the UHPLC-QTOF/MS Method

The overlapping total ion current (TIC) chromatograms (**Supplementary Figure S3**) of QC samples demonstrated the acceptable variations occurred during the large-scale sample analysis. Meantime, six selected extracted ion chromatograms (EICs) in QC samples were used to assess the system repeatability and stability. Taking tissue samples in positive mode as an example, the retention times, peak areas, and mass accuracies of these six selected peaks showed acceptable RSDs. RSDs of these six peaks were 0.00000–2.81406% for retention times, 7.13116–17.37194% for peak areas, and 0.00008–0.07090% for mass accuracies (**Supplementary Tables S1**, **S2**).

### Metabolic Profiles of the Control and CKD Rats

**Figure 3:**
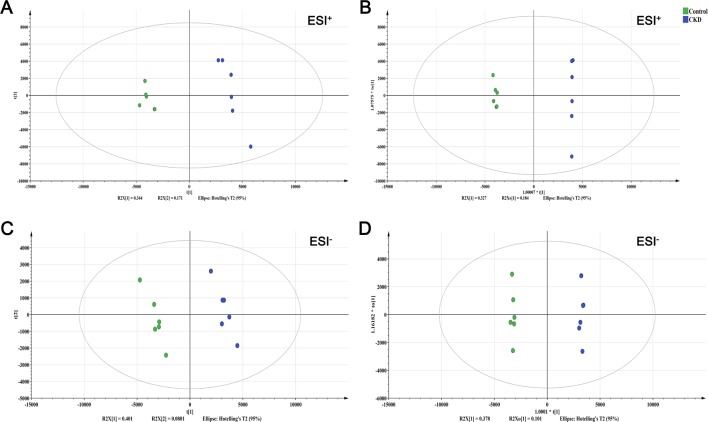
The score plots from PCA and OPLS-DA model. **(A)** PCA-X score plot of positive ion mode. **(B)** OPLS-DA score plot of positive ion mode. **(C)** PCA-X score plot of negative ion mode. **(D)** OPLS-DA score plot of negative ion mode. ESI^+^ means the positive electrospray ionization. ESI^−^ means the negative electrospray ionization. Green circle corresponds to the control group; blue circle corresponds to the CKD group.

To evaluate the alterations of metabolites in CKD rats, PCA and OPLS-DA were performed using data from the control and CKD groups. PCA score plots showed the obvious separation trend between the control and CKD group (**Figures 3A, C**), indicating that renal metabolic states of CKD rats were significantly changed in relative to control rats. OPLS-DA score plots could also readily be divided into two clusters (**Figures 3B, D**). These results illustrated that the metabolites in the control and CKD groups had been completely separated either the positive or the negative ion mode.

### Identification of Differential Metabolites in CKD Rats

**Figure 4:**
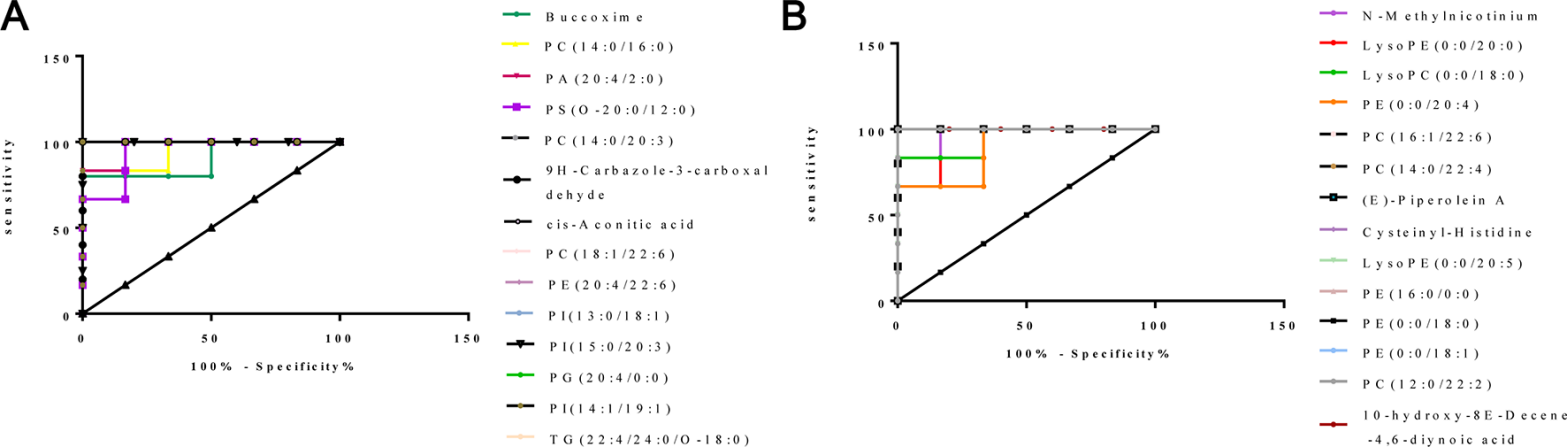
ROC curves of 28 potential biomarkers from kidney in CKD rats. **(A)** ROC analysis of 14 upregulated biomarkers. **(B)** ROC analysis of 14 downregulated biomarkers.

In total, 28 metabolites contributing to CKD phenotype were found and identified in the kidney (**Table 2**). The ROC curve was performed to evaluate the predictive value of these screened potential biomarkers. The 14 upregulated metabolites provided good diagnostic abilities with average area under the curve (AUC) at 0.9000–1.0000 and *p* value at 0.0016–0.0163 (**Figure 4A**, **Supplementary Table S3**). The 14 downregulated metabolites also provided good diagnostic abilities with AUC at 0.8333–1.0000 and *p* value at 0.0039–0.0250 (**Figure 4B**, **Supplementary Table S4**). The results showed that the identified biomarkers had a powerful diagnostic performance.

**Table 2:** Identities of differential metabolites between control and CKD model.

NO.	Abbreviation	Molecular formula	m/z	Rt (min)	Matched Mass	ppm	VIP	p value	Fold
1	N-Methylnicotinium	C_11_H_17_N_2_	221.1033	2.84	221.1031	0.9	185	0.0065	0.40
2	Buccoxime	C_10_H_17_NO	150.1277	27.27	150.1283	-4.0	3.18	0.0163	1.16
3	PC (16:1/22:6)	C_46_H_78_NO_8_P	804.5507	20.6	804.5538	-3.9	1.32	0.0039	0.25
4	PC (14:0/22:4)	C_44_H_80_NO_8_P	782.5697	27.34	782.5694	0.4	9.91	0.0039	0.58
5	PC (14:0/20:3)	C_42_H_78_NO_8_P	756.5526	27.35	756.5538	-1.6	1.93	0.0039	2.29
6	(E)-Piperolein A	C_19_H_25_NO_3_	104.0537	1.17	104.0539	1.9	1.09	0.0039	0.42
7	Cysteinyl-Histidine	C_9_H_14_N_4_O_3_S	279.0541	1.27	279.0533	2.9	1.08	0.0062	0.27
8	9H-Carbazole-3-carboxaldehyde	C_13_H_9_NO	214.0665	1.17	214.0668	1.4	1.18	0.0039	6.46
9	cis-Aconitic acid	C_6_H_6_O_6_	173.01	2.96	173.0092	4.6	2.03	0.0039	29.92
10	PC (14:0/16:0)	C_38_H_76_NO_8_P	750.5287	27.6	750.5291	0.5	2.07	0.0163	2.37
11	PC (18:1/22:6)	C_48_H_82_NO_8_P	830.5711	27.6	830.5705	0.7	2.67	0.0039	3.42
12	PA (20:4/2:0)	C_25_H_43_O_7_P	531.2737	12.23	531.2728	1.7	1.01	0.0065	13.54
13	LysoPE (0:0/20:5)	C_25_H_42_NO_7_P	544.2682	9.67	544.2681	0.2	1.21	0.0039	0.04
14	LysoPE (0:0/20:0)	C_25_H_52_NO_7_P	508.3433	16.99	508.3409	4.7	3.61	0.0163	0.54
15	PE (20:4/22:6)	C_47_H_74_NO_8_P	810.5102	5028	810.5079	2.8	9.65	0.0039	7.54
16	LysoPC (0:0/18:0)	C_26_H_54_NO_7_P	568.3604	16.99	568.362	2.8	3.55	0.0104	0.53
17	PE (16:0/0:0)	C_21_H_44_NO_7_P	452.2841	12.5	452.2783	12.8	2.87	0.0039	0.28
18	PE (0:0/18:0)	C_23_H_48_NO_7_P	480.3138	16.84	480.3096	8.8	4.13	0.0039	0.44
19	PE (0:0/20:4)	C_25_H_44_NO_7_P	500.2813	10.19	500.2783	6.0	3.47	0.0250	0.54
20	PE (0:0/18:1)	C_23_H_46_NO_7_P	524.3009	17.03	524. 2994	2.9	2.88	0.0039	0.31
21	10-hydroxy-8E-Decene-4,6-diynoic acid	C_10_H_10_O_3_	177.059	1.2	177.0557	18.6	1.61	0.0039	0.07
22	PI (13:0/18:1)	C_40_H_75_O_13_P	793.4846	27.6	793.4873	3.4	2.04	0.0039	3.30
23	PI (15:0/20:3)	C_44_H_79_O_13_P	845.5125	27.6	845.5186	7.2	1.89	0.0039	16.80
24	PG (20:4/0:0)	C_26_H_45_O_9_P	531.2737	12.23	531.2729	1.5	1.01	0.0065	13.54
25	PS (O-20:0/12:0)	C_38_H_76_NO_9_P	766.5232	5.36	766.524	1.0	9.08	0.0104	3.44
26	PI (14:1/19:1)	C_42_H_77_O_13_P	819.4969	27.6	819.5029	7.3	1.76	0.0039	2.34
27	TG (22:4/24:0/O-18:0)	C_67_H_124_O_5_	335.3083	27.17	335.3077	1.8	1.30	0.0039	10.58
28	PC (12:0/22:2)	C_42_H_80_NO_8_P	758.5695	20.43	758.5694	0.13	9.85	0.0039	0.57

LysoPC, lysophosphatidylcholines; LysoPE, lysophosphatidylethanolamine; PA, phosphatidic acids; PC, phosphatidylcholines; PE, phosphatidylethanolamines; PG, phosphoglycerols; PI, phosphatidylinositols; PS, phosphatidylserines; TG, triglyceride.

### Metabolic Pathway Analysis

**Figure 5:**
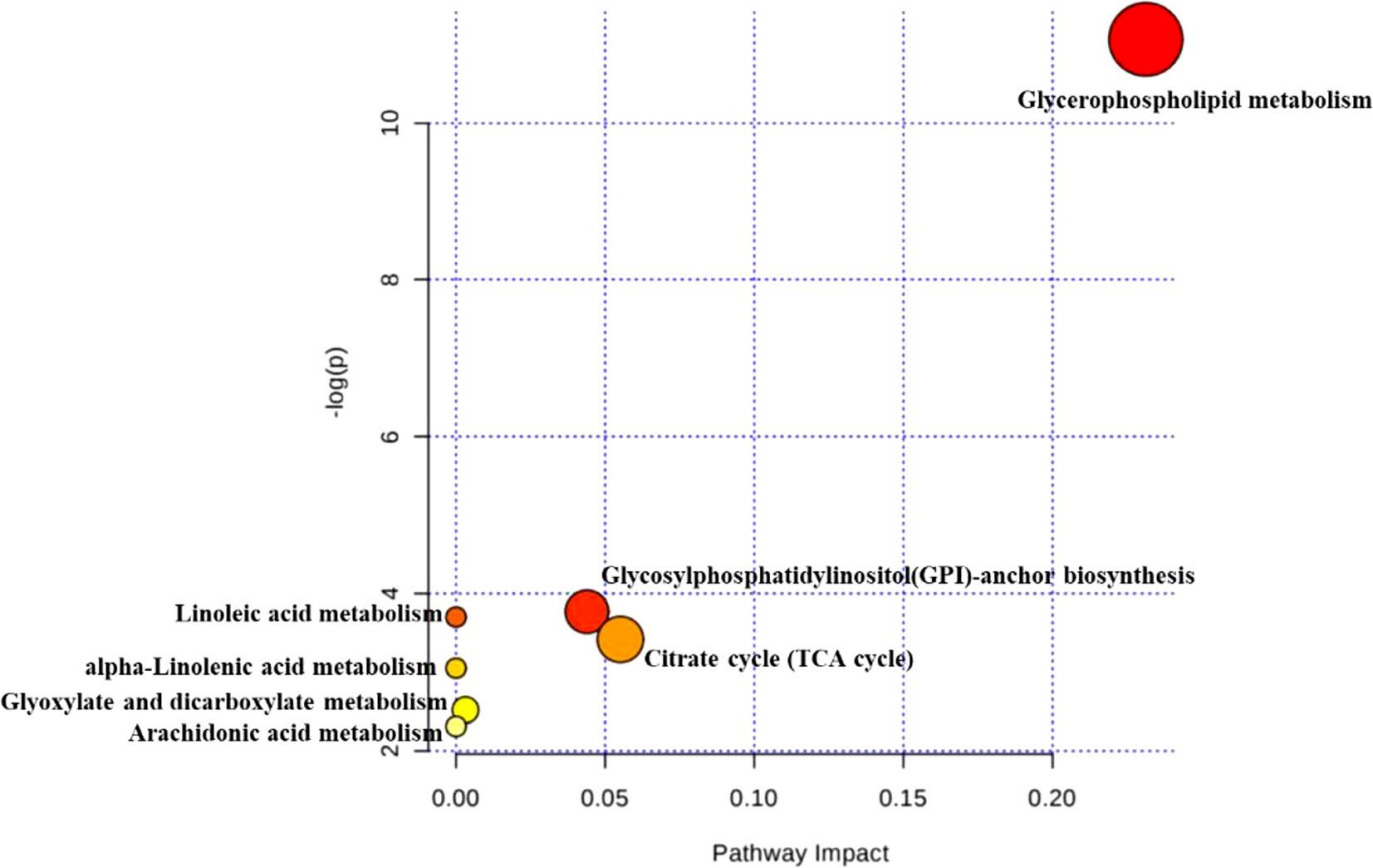
Disordered pathways in the CKD group. The topology map generated from MetaboAnalyst described the impact of 28 metabolites identified between control and CKD groups on metabolic pathway.

According to MetaboAnalyst database analysis, there were seven main metabolic pathways disordered in CKD rats, which were glycerophospholipid metabolism, glycosylphosphatidylinositol (GPI)-anchor biosynthesis, citrate cycle (TCA cycle), linoleic acid metabolism, alpha-linolenic acid metabolism, glyoxylate and dicarboxylate metabolism, and arachidonic acid metabolism (**Figure 5**).

### HDD Altered Metabolic Profiles in CKD Rats

**Figure 6:**
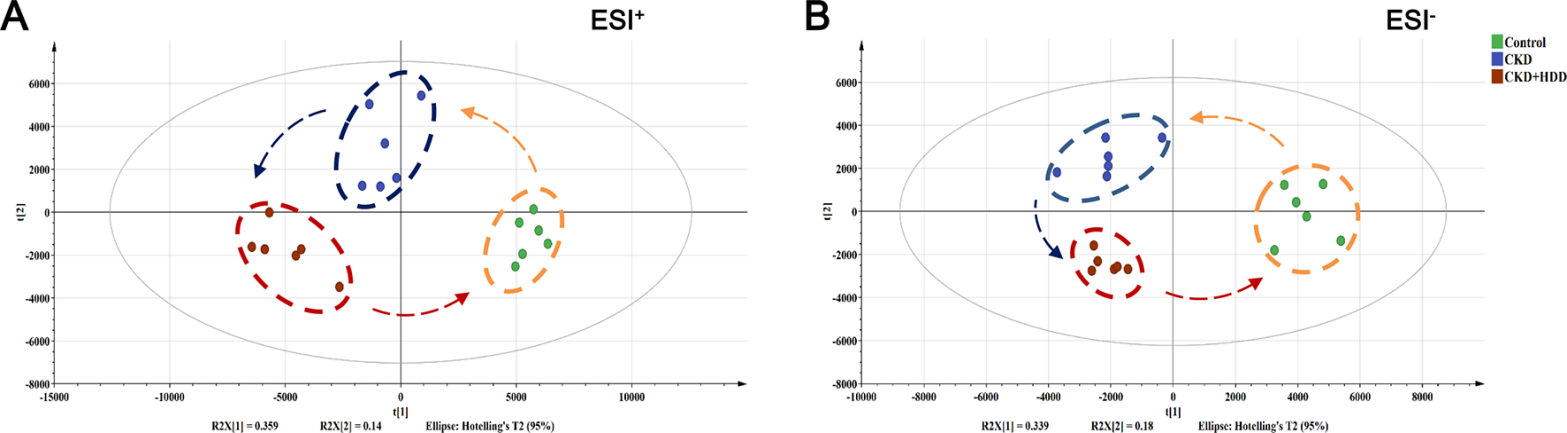
Effect of HDD on the metabolic profiling by PLS-DA score plots. **(A)** PLS-DA score plot of positive ion mode. **(B)** PLS-DA score plot of negative ion mode.

**Figure 7:**
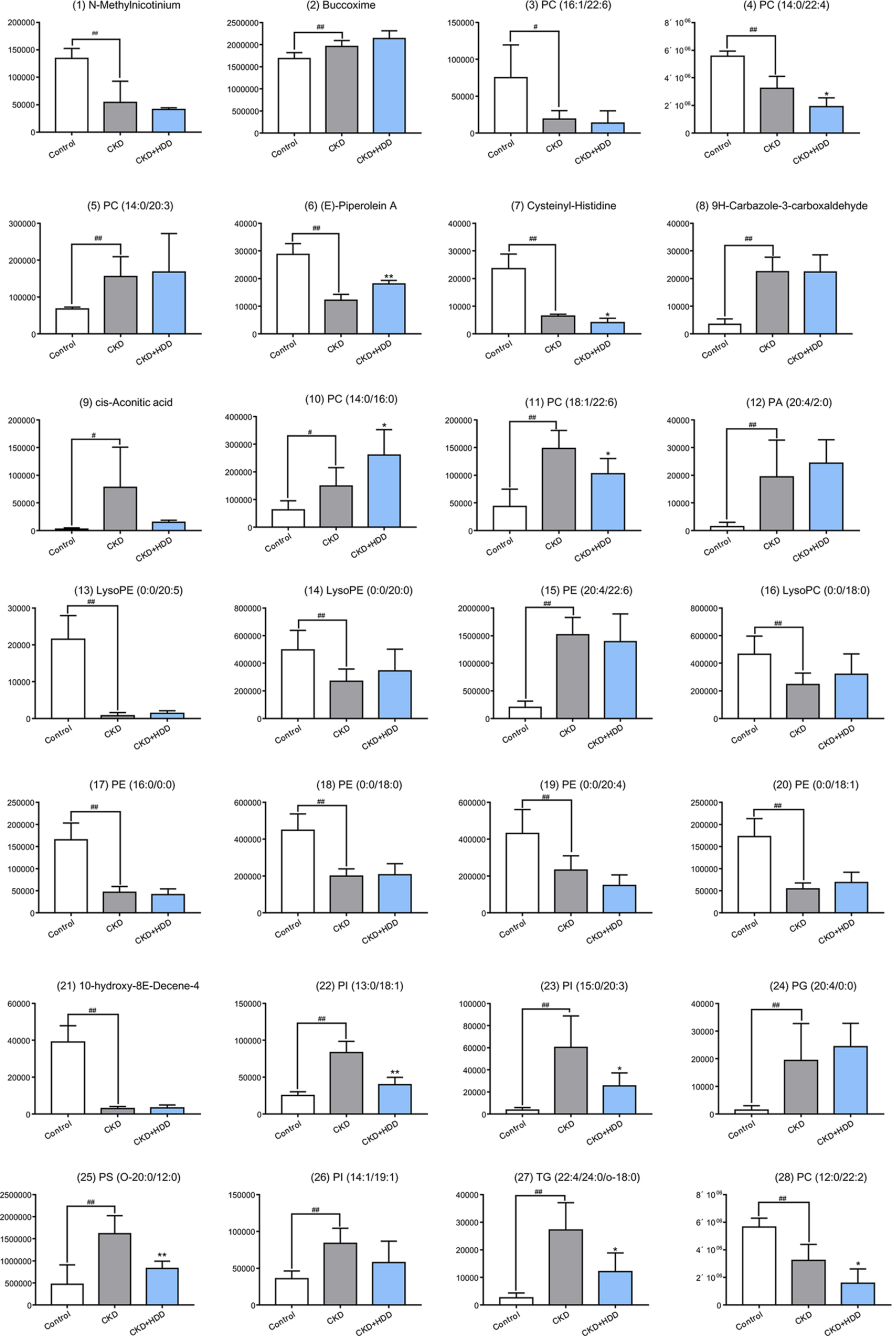
The relative content of 28 metabolites in the control, CKD, and CKD + HDD group. Data are represented as means ± SD, n ≥ 5 rats per group (*P < 0.05, **P < 0.01 compared with the CKD group; ^#^*P* < 0.05, ^##^*P* < 0.01 compared with the control group).

In order to reveal the effect of HDD on metabolic profiles in CKD rats, PLS-DA analysis was performed to obtain the changes in metabolic trajectory. The PLS-DA score plot showed that the three groups of renal samples were obviously divided into three categories, and the metabolic trajectory of the CKD+HDD group deviated from the CKD group and moved to the control group in both positive and negative ion modes (**Figure 6**), which indicated that renal metabolites were obviously changed and had a tendency to recover after HDD treatment. **Figure 7** illustrated the levels of 14 upregulated biomarkers and 14 downregulated biomarkers among the control group and CKD group and CKD + HDD group. After HDD treatment, the levels of (E)-Piperolein A, phosphatidylcholines (PC) (18:1/22:6), phosphatidylinositols (PI) (13:0/18:1), PI (15:0/20:3), phosphatidylserines (PS) (O-20:0/12:0), and triglyceride (TG) (22:4/24:0/O-18:0) were reversed and restored to near control level.

## Discussion

The present study described a renal metabolomic evaluation of CKD rat model induced by adenine and the treatment of HDD based on UHPLC-QTOF/MS metabolomics approach. HDD markedly reduced serum levels of creatinine and BUN, and ameliorated tubular atrophy and interstitial fibrosis in CKD rats. Furthermore, metabolomics analysis indicated that HDD effectively regulated the perturbed metabolism in the kidney of CKD rats.

CKD is a global public health problem, but its pathogenesis, early diagnosis, and treatment are still not satisfactory. Metabolomics is defined as “the quantitative measurement of the dynamic multiparametric metabolic response of living systems to pathophysiological stimuli or genetic modification” (Nicholson et al., 1999; Fiehn, 2002; Nicholson et al., 2002). Metabolites are the final product of cellular metabolism and can be thought of as the ultimate response of the system to heredity and environment. The use of metabolomics methods to study the metabolite composition of the system will provide a powerful tool for understanding the pathological mechanisms of CKD and developing new treatment strategies for treatment (Rhee, 2015; Grams et al., 2018). A series of experimental and clinical studies have been conducted to investigate the metabolic profiles of serum, plasma, urine, or tissue in CKD rodent models or CKD patients and have identified several metabolomics biomarkers and pathways (Zhao, 2013; Hocher and Adamski, 2017; Kalim and Rhee, 2017; Chen et al., 2019). Although TCM has been effective in treating CKD, it lacks the necessary clear molecular mechanisms. Consistent with the overall thinking of TCM, metabolomics has potential for TCM bioactivity and mechanism evaluation (Wang et al., 2017a). Many research groups have used metabolomics to evaluate efficacy and mechanism of TCM in CKD animal models using pure compounds from TCM (Zhao et al., 2014) or TCM extract (Zhang et al., 2016). In the present study, we identified 28 significant changed metabolites in the kidney of CKD rats, most of which were lipids (PCs, PEs, PIs, LysoPCs, or LysoPEs). Behind renopretective effect in CKD, HDD showed a regulatory effect on kidney metabolites, which mainly focused on glycerophospholipid metabolism (PC, PI, and PS). These findings might provide important clues for further mechanism studies.

Glycerophospholipids, glycerol-based phospholipids, are the main component of biological membranes and play a major role in cell signal induction and transport (Hermansson et al., 2011). Our results showed that glycerophospholipid metabolism is the most significantly altered pathway in the kidney of CKD rats and is a major aspect of metabolite regulation in HDD treatment response. Previous studies have shown the presence of glycerophospholipid abnormalities in CKD patients and animal models (Zhao et al., 2015c; Barrios et al., 2016; Ye and Mao, 2016). Nkuipou-Kenfack et al. reported abnormal PC metabolism in mild to advanced CKD patients (Nkuipou-Kenfack et al., 2014). Chen et al. included 180 patients with advanced CKD and found significant increase in the levels of glycerophospholipids which inversely correlated with the level of estimated glomerular filtration rate (eGFR) (Chen et al., 2017). These studies collectively suggest an important role for glycerophospholipids in CKD progression.

According to TCM theory, Qi deficiency and blood stasis (Qi-Xu-Xue-Yu) runs through the CKD process (Liu et al., 2019). HDD is composed of Astragali Radix (Huang-qi) served as replenishing Qi and Salviae Miltiorrhizae Radix et Rhizoma (Dan-shen) served as activating blood, and is one of the most common used drug pair in clinical treatment of CKD. In this study, metabolomics analysis indicated that renal metabolites had a tendency to recover in CKD rats after HDD treatment. Previous studies have reported the effects of Huang-qi or Dan-shen on metabolic pathways in different disease models. Li et al. found that the antifatigue effect of Huang-qi was associated with regulating glycometabolism, lipid metabolism, and energy metabolism (Li et al., 2014). Urinary metabolomics revealed that Huang-qi injections could regulate amino acid metabolism, TCA cycle, fatty acid metabolism, vitamin B6 metabolism, and purine metabolism in cisplatin-induced nephrotoxic rats (Li et al., 2017). Zhang et al. reported that the protective mechanism of Dan-shen in Alzheimer’s disease was related to glutathione metabolism, phenylalanine tyrosine and tryptophan biosynthesis, TCA cycle, glycerophospholipid metabolism, etc. (Zhang et al., 2019). Although the bioactive components of HDD and their interactions were obscure, metabolomics shed light on offering potential biomarkers and metabolic patterns, which will benefit drug efficacy evaluation and potential mechanism research.

## Conclusion

In this study, UHPLC-QTOF/MS-based nontargeted metabolomic approach was applied to investigate the protective effects of HDD against adenine-induced CKD in rats. Twenty-eight metabolites contributing to CKD phenotype were identified in the kidney. The primary metabolic pathways disordered in the kidney of CKD rats were glycerophospholipid metabolism, GPI-anchor biosynthesis, and TCA cycle. Treatments with HDD attenuated kidney injury, improved renal function, and partially reversed abnormalities of renal metabolome.

## Data Availability

The datasets generated for this study are available on request to the corresponding author.

## Ethics Statement

The animal study was reviewed and approved by Ethics Committee of Shenzhen Traditional Chinese Medicine Hospital, Guangzhou University of Chinese Medicine. 

## Author Contributions

XL and SL conceived and designed the experiments. LZ and JC performed herbal preparation. BZ, SH, and FW carried out animal experiment and conducted the pathological analysis. JL, YZ, and LZ contributed to data collection and manuscript review. XL, BZ, and JC performed the experiments, analyzed the data, prepared figures, and wrote the manuscript. All authors have read and approved the manuscript.

## Funding

This study was supported by Shenzhen Science and Technology Plan Project (JCYJ20170307154652899 and ZDSYS201606081515458), Natural Science Foundation of China (81603437 and 81804052), Natural Science Foundation of Guangdong Province (2018A030313305), and Shenzhen Municipal Health Commission (SZLY2018005).

## Conflict of Interest Statement

The authors declare that the research was conducted in the absence of any commercial or financial relationships that could be construed as a potential conflict of interest.

## Footnotes

1 https://metlin.scripps.edu

2 http://www.hmdb.ca

3 https://www.genome.jp/kegg

4 https://www.metaboanalyst.ca
