# Supplementary material for: Metabolomics Analysis Reveals the Protection Mechanism of Huangqi–Danshen Decoction on Adenine-Induced Chronic Kidney Disease in Rats
Source: Front Pharmacol. 2019 Sep 10;10:992. doi: 10.3389/fphar.2019.00992 (PMC6747014; doi:10.3389/fphar.2019.00992)
Supplement: Supplementary file 1 [file DataSheet_1.docx]

**Supplementary Figures**


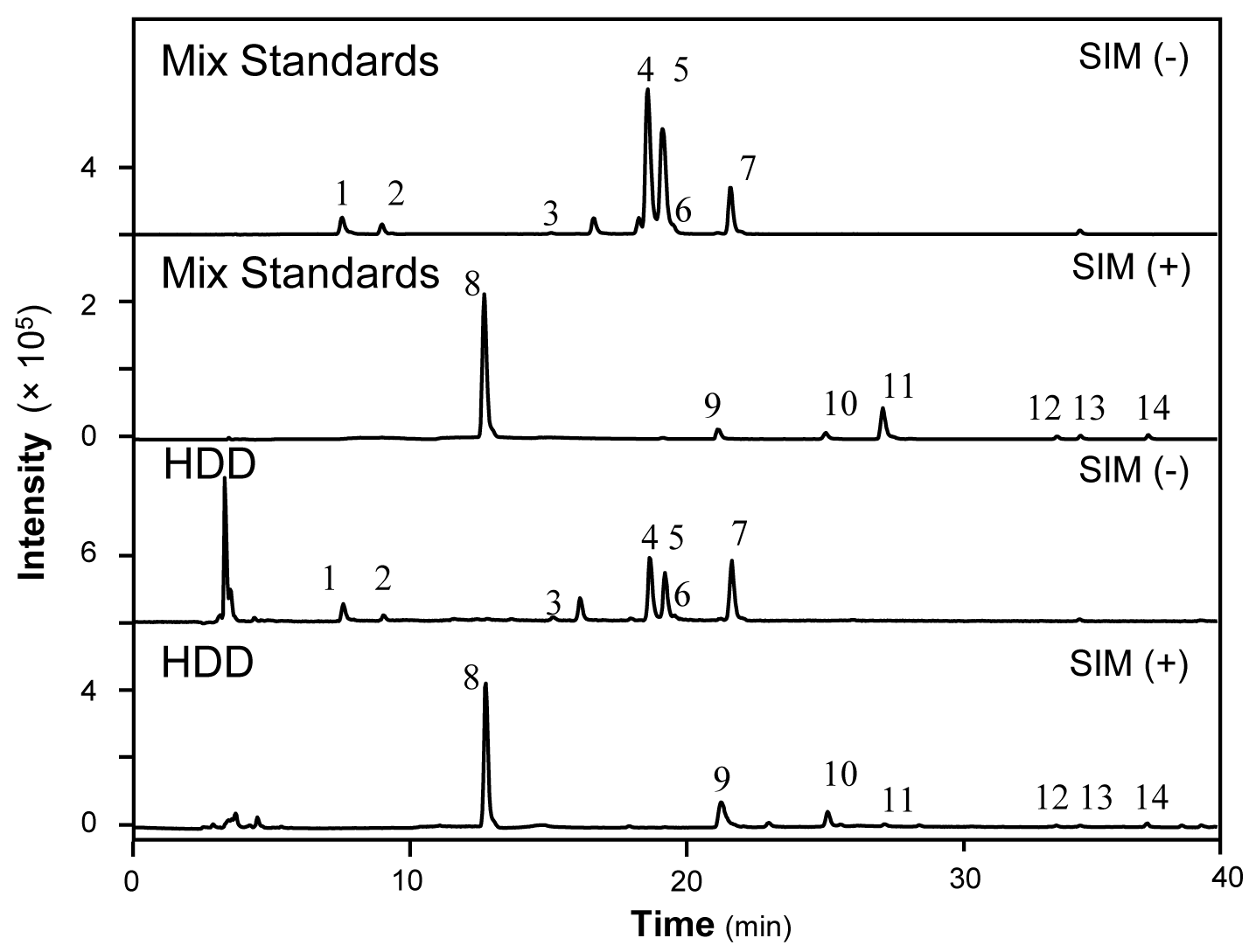


**Supplementary Figure S1. HPLC-MS chromatogram of HDD.**

Equipment type: Shimadzu LC-20AT; chromatographic column: ODS HYPERSIL DIM (mm) (250×4.6 mm,3 μm; flow rate: 0.8 ml/min; mobile phase condition: the proportion of 0.01% formic acid (A) and acetonitrile (B) was as follows: 0~15 min, 18%~28% B; 15~29 min, 28%~42% B; 29~33 min, 42%~52% B; 33~37 min, 52%~60% B; 37~41 min, 60%~90% B; A Shimadzu mass spectrum (LCMS-2020) equipped with an electrospray ionisation (ESI) ion source was operated in positive and negative modes, and the selected ion monitoring (SIM) was used. Optimized mass spectra were acquired with an interface voltage of 4.5 kV. Nitrogen was used as nebulizer gas at a flow rate of 1.5 L/min and dry gas flow of 15 L/min. Labsolution workstation software was used for data acquisition and processing. The denotation peaks 1-14: (1) Protocatechualdehyde, (2) Ferulic Acid (internal standard, IS), (3) Caffeic Acid, (4) Rosmarinic Acid, (5) Lithospermic Acid, (6) Notoginsenoside R1 (IS), (7) Ononin, (8) Calycosin 7-O-beta-D-glucoside, (9) Salvianolic Acid B, (10) Daidzein (IS), (11) Calycosin, (12) Astragaloside IV, (13) Astragaloside III, (14) Astragaloside II.


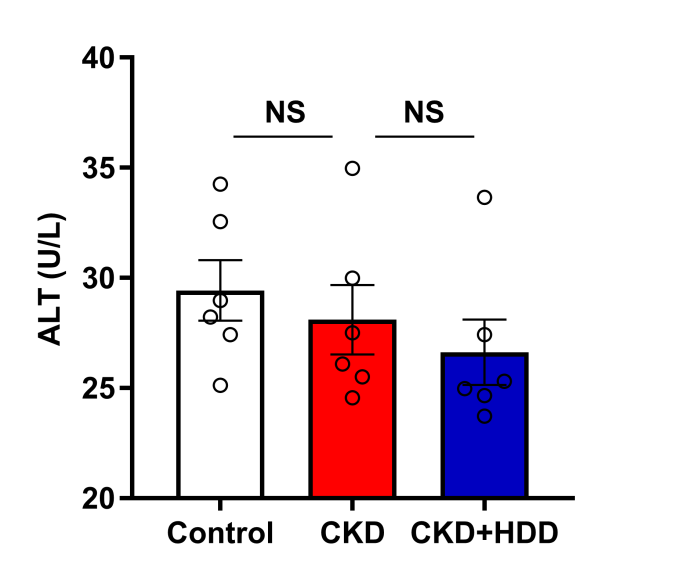

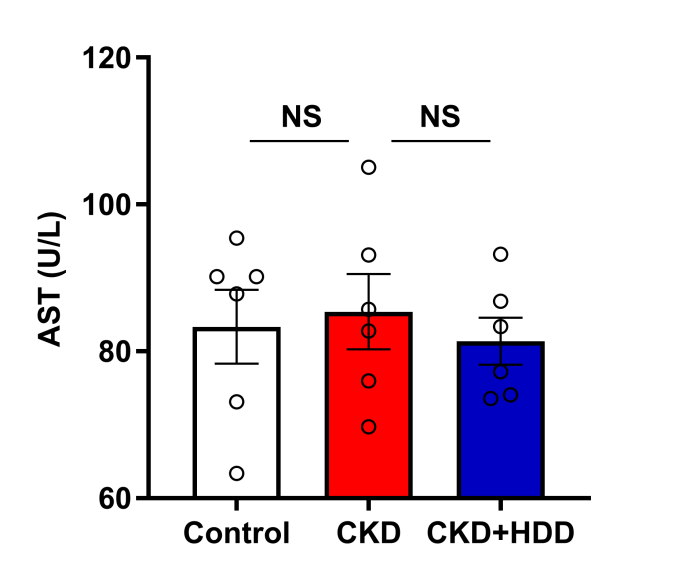


**B**

**A**

**Supplementary Figure S2. HDD has no effect on liver function.** (**A**) Serum aspartate transaminase (AST) levels. (**B**) Serum alanine transaminase (ALT) levels. Data are presented as the means ± SEM, n=6 rats per group.

**A**


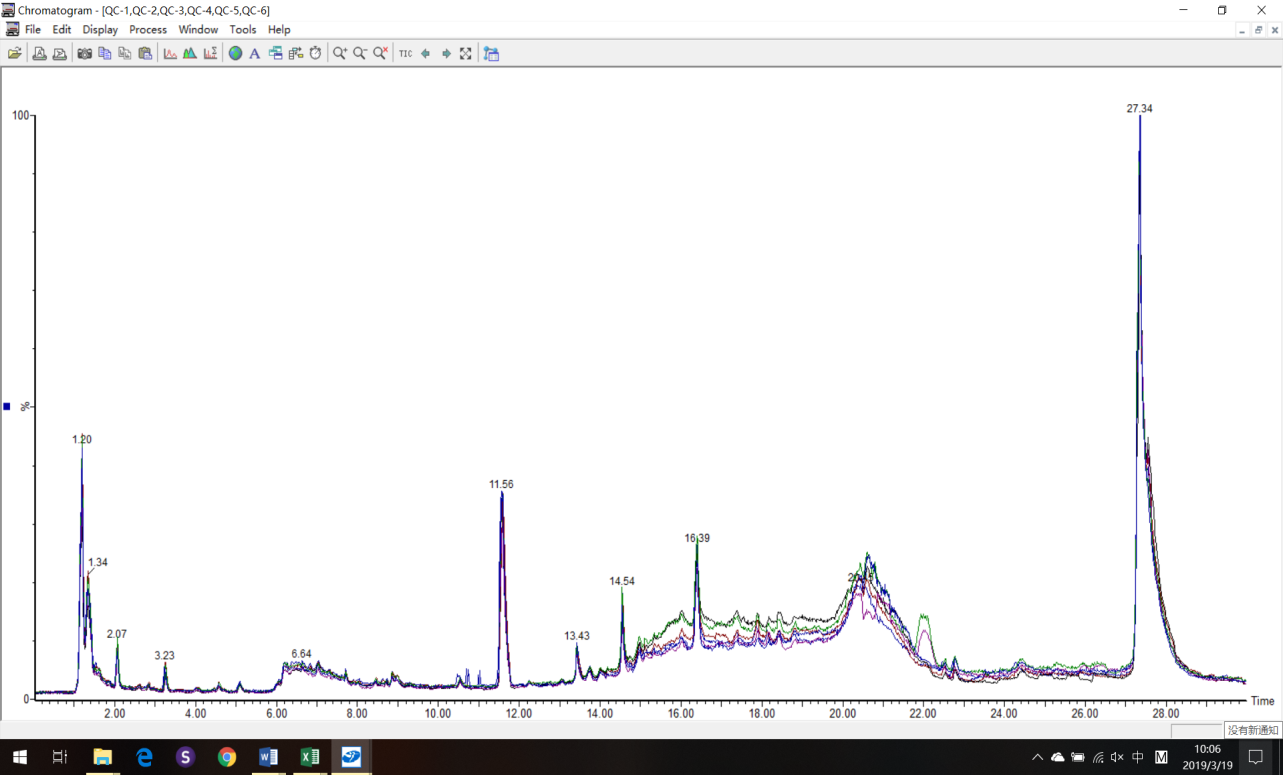


**B**


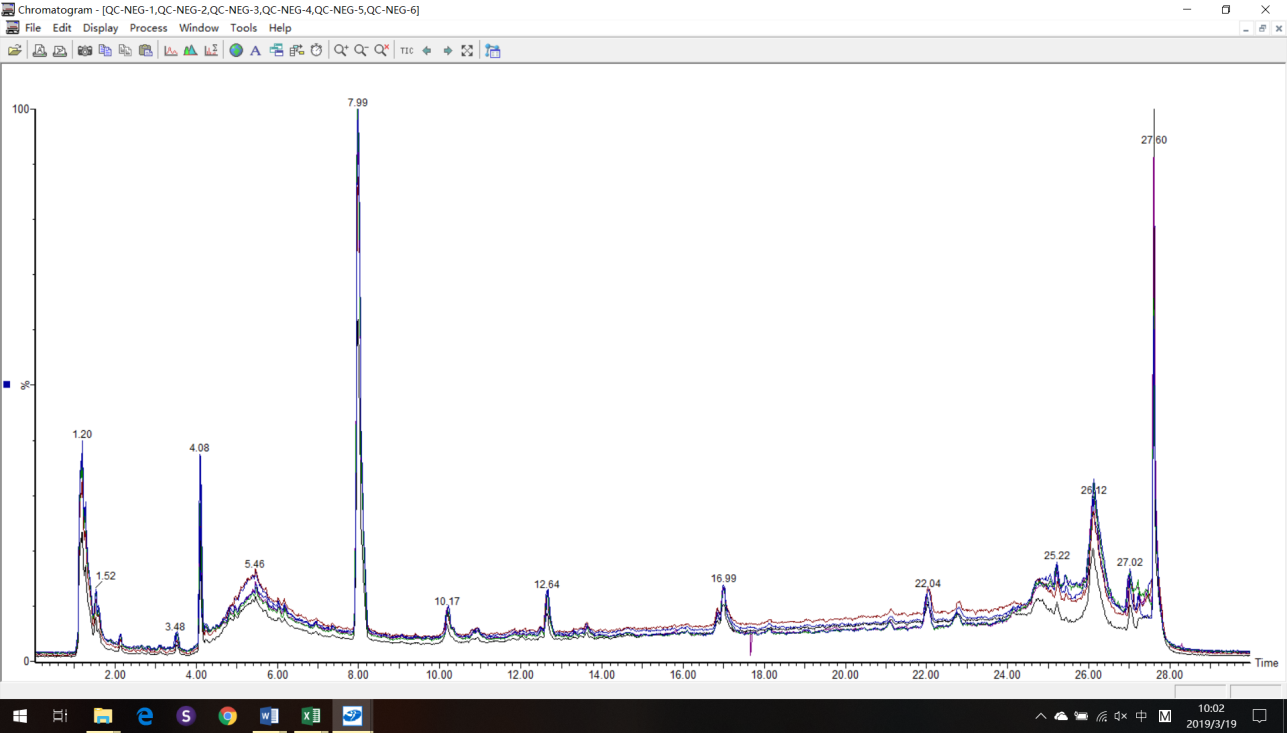


**Supplementary Figure S3. The overlapping typical total ion chromatograms (TICs) of QC samples obtained from LC-MS in positive and negative modes.** (**A**) Six QC samples from tissue in positive mode. (**B**) Six QC samples from tissue in negative mode.

**Supplementary Tables**

**Supplementary Table S1. Six extracted ions in six QC samples from tissue in positive mode.**

| **Extracted Ions** | **No.** | **Retention time** | **Peak area** | **Mass accuracies** |
| --- | --- | --- | --- | --- |
| 432.3472 | 1 | 6.516 | 175.52 | 432.3463 |
|  | 2 | 6.516 | 179.92 | 432.3468 |
|  | 3 | 6.516 | 168.82 | 432.3463 |
|  | 4 | 6.516 | 167.49 | 432.3473 |
|  | 5 | 6.516 | 227.63 | 432.3478 |
|  | 6 | 6.516 | 195.27 | 432.3474 |
|  | RSD(%) | 0.00000 | 12.28248 | 0.00014 |
| 446.3267 | 1 | 6.481 | 297.85 | 446.3265 |
|  | 2 | 6.482 | 326.45 | 446.3267 |
|  | 3 | 6.482 | 352.69 | 446.3266 |
|  | 4 | 6.482 | 329.05 | 446.3273 |
|  | 5 | 6.842 | 403.11 | 446.3264 |
|  | 6 | 6.841 | 396.66 | 446.3263 |
|  | RSD(%) | 2.81406 | 11.89379 | 0.00008 |
| 574.3741 | 1 | 6.704 | 30.36 | 574.373 |
|  | 2 | 6.704 | 32.31 | 574.3766 |
|  | 3 | 6.704 | 30.1 | 574.3703 |
|  | 4 | 6.704 | 38.14 | 574.3773 |
|  | 5 | 6.704 | 32.55 | 574.3735 |
|  | 6 | 6.703 | 36.48 | 573.377 |
|  | RSD(%) | 0.00609 | 9.85840 | 0.07090 |
| 661.4057 | 1 | 6.197 | 44.81 | 661.4094 |
|  | 2 | 6.198 | 39.18 | 661.4093 |
|  | 3 | 6.198 | 36.45 | 661.3966 |
|  | 4 | 6.198 | 39.81 | 661.4146 |
|  | 5 | 6.198 | 47.41 | 661.4067 |
|  | 6 | 6.197 | 57.62 | 661.4078 |
|  | RSD(%) | 0.00833 | 17.38194 | 0.00090 |
| 531.3429 | 1 | 6.464 | 61.59 | 531.3403 |
|  | 2 | 6.465 | 60.59 | 531.3375 |
|  | 3 | 6.465 | 60.92 | 531.3427 |
|  | 4 | 6.465 | 63.87 | 531.3427 |
|  | 5 | 6.465 | 83.99 | 531.3442 |
|  | 6 | 6.464 | 59.88 | 531.3408 |
|  | RSD(%) | 0.00799 | 14.33076 | 0.00045 |
| 608.3797 | 1 | 6.180 | 52.54 | 608.3796 |
|  | 2 | 6.181 | 53.36 | 608.3772 |
|  | 3 | 6.181 | 58.48 | 608.3801 |
|  | 4 | 6.181 | 59.27 | 608.3817 |
|  | 5 | 6.181 | 53.52 | 608.3766 |
|  | 6 | 6.18 | 62.46 | 608.377 |
|  | RSD(%) | 0.00836 | 7.13116 | 0.00034 |

**Supplementary Table S2. Six extracted ions in six QC samples from tissue in negative mode.**

| **Extracted Ions** | **No.** | **Retention time** | **Peak area** | **Mass accuracies** |
| --- | --- | --- | --- | --- |
| 365.1468 | 1 | 1.521 | 165.04 | 365.1461 |
|  | 2 | 1.521 | 177.51 | 365.1466 |
|  | 3 | 1.521 | 178.95 | 365.1458 |
|  | 4 | 1.521 | 173.98 | 365.1469 |
|  | 5 | 1.521 | 194.61 | 365.1469 |
|  | 6 | 1.521 | 181.8 | 365.1464 |
|  | RSD(%) | 0.00000 | 5.44479 | 0.00012 |
| 465.3098 | 1 | 27.587 | 1651.49 | 465.3104 |
|  | 2 | 27.586 | 1602.94 | 465.3097 |
|  | 3 | 27.586 | 1967.51 | 465.3097 |
|  | 4 | 27.586 | 1832.42 | 465.3097 |
|  | 5 | 27.586 | 1267.95 | 465.3098 |
|  | 6 | 27.587 | 1199.1 | 465.3102 |
|  | RSD(%) | 0.00187 | 19.14876 | 0.00007 |
| 163.0588 | 1 | 3.334 | 418.47 | 163.0585 |
|  | 2 | 3.334 | 424 | 163.058 |
|  | 3 | 3.334 | 419.85 | 163.0586 |
|  | 4 | 3.334 | 431.52 | 163.0582 |
|  | 5 | 3.334 | 387.1 | 163.0583 |
|  | 6 | 3.334 | 443.22 | 163.0575 |
|  | RSD(%) | 0.00000 | 4.47206 | 0.00024 |
| 359.3069 | 1 | 26.314 | 1005.85 | 359.3081 |
|  | 2 | 26.313 | 931.97 | 359.3071 |
|  | 3 | 26.313 | 949.27 | 359.3074 |
|  | 4 | 26.313 | 830.81 | 359.3073 |
|  | 5 | 26.313 | 875.58 | 359.3067 |
|  | 6 | 26.314 | 1010.15 | 359.3072 |
|  | RSD(%) | 0.00196 | 7.61044 | 0.00013 |
| 331.2772 | 1 | 25.075 | 1813.5 | 331.2771 |
|  | 2 | 25.075 | 1723.22 | 331.2757 |
|  | 3 | 25.075 | 1789.71 | 331.2761 |
|  | 4 | 25.075 | 867.71 | 331.2774 |
|  | 5 | 25.075 | 1104.29 | 331.2772 |
|  | 6 | 25.075 | 1515.96 | 331.2782 |
|  | RSD(%) | 0.00000 | 26.93617 | 0.00027 |
| 134.0799 | 1 | 3.515 | 702.78 | 134..0792 |
|  | 2 | 3.515 | 697.52 | 134.0792 |
|  | 3 | 3.515 | 689.48 | 134.0797 |
|  | 4 | 3.515 | 763.07 | 134.0799 |
|  | 5 | 3.515 | 756.56 | 134.0799 |
|  | 6 | 3.516 | 826.24 | 134.0798 |
|  | RSD(%) | 0.01161 | 7.15329 | 0.00022 |

**Supplementary Table S3. ROC curve analysis of 14 up-regulated biomarkers from tissue.**

| Metabolites | CKD vs Control AUCs | Std. Error^a^ | p value | Asymptotic 95% Confidence Interval | |
| --- | --- | --- | --- | --- | --- |
|  |  |  |  | Lower Bound | Upper Bound |
| Buccoxime | 0.9000 | 0.0719 | 0.0016 | 0.7591 | 1.0410 |
| PC (14:0/20:3) | 1.0000 | 0.0000 | 0.0143 | 1.0000 | 1.0000 |
| 9H-Carbazole-3-carboxaldehyde | 1.0000 | 0.0000 | 0.0039 | 1.0000 | 1.0000 |
| cis-Aconitic acid | 1.0000 | 0.0000 | 0.0062 | 1.0000 | 1.0000 |
| PC (14:0/16:0) | 0.9167 | 0.0814 | 0.0163 | 0.7572 | 1.0760 |
| PC (18:1/22:6) | 1.0000 | 0.0000 | 0.0039 | 1.0000 | 1.0000 |
| PA (20:4/2:0) | 0.9722 | 0.0427 | 0.0065 | 0.8886 | 1.0560 |
| PE (20:4/22:6） | 1.0000 | 0.0000 | 0.0039 | 1.0000 | 1.0000 |
| PI (13:0/18:1） | 1.0000 | 0.0000 | 0.0039 | 1.0000 | 1.0000 |
| PI (15:0/20:3) | 1.0000 | 0.0000 | 0.0039 | 1.0000 | 1.0000 |
| PG (20:4/0:0) | 1.0000 | 0.0000 | 0.0062 | 1.0000 | 1.0000 |
| PS (O-20:0/12:0) | 0.9444 | 0.0668 | 0.0104 | 0.8136 | 1.0750 |
| PI (14:1/19:1) | 1.0000 | 0.0000 | 0.0039 | 1.0000 | 1.0000 |
| TG (22:4/24:0/O-18:0) | 1.0000 | 0.0000 | 0.0039 | 1.0000 | 1.0000 |

**Supplementary Table S4. ROC curve analysis of 14 down-regulated biomarkers from tissue.**

| Metabolites | CKD vs Control AUCs | Std. Error^a^ | p value | Asymptotic 95% Confidence Interval | |
| --- | --- | --- | --- | --- | --- |
|  |  |  |  | Lower Bound | Upper Bound |
| N-Methylnicotinium | 0.9722 | 0.0427 | 0.0065 | 0.8886 | 1.0560 |
| PC (16:1/22:6) | 1.0000 | 0.0000 | 0.0062 | 1.0000 | 1.0000 |
| PC (14:0/22:4) | 1.0000 | 0.0000 | 0.0039 | 1.0000 | 1.0000 |
| (E)-Piperolein A | 1.0000 | 0.0000 | 0.0039 | 1.0000 | 1.0000 |
| Cysteinyl-Histidine | 1.0000 | 0.0000 | 0.0062 | 1.0000 | 1.0000 |
| LysoPE (0:0/20:5) | 1.0000 | 0.0000 | 0.0062 | 1.0000 | 1.0000 |
| LysoPE (0:0/20:0) | 0.9167 | 0.0814 | 0.0163 | 0.7572 | 1.0760 |
| LysoPC (0:0/18:0) | 0.9444 | 0.0668 | 0.0104 | 0.8136 | 1.0750 |
| PE (16:0/0:0) | 1.0000 | 0.0000 | 0.0039 | 1.0000 | 1.0000 |
| PE (0:0/18:0) | 1.0000 | 0.0000 | 0.0039 | 1.0000 | 1.0000 |
| PE (0:0/20:4) | 0.8889 | 0.0980 | 0.0250 | 0.6968 | 1.0810 |
| PE (0:0/18:1) | 1.0000 | 0.0000 | 0.0039 | 1.0000 | 1.0000 |
| 10-hydroxy-8E-Decene-4,6-diynoic acid | 1.0000 | 0.0000 | 0.0039 | 1.0000 | 1.0000 |
| PC (12:0/22:2) | 1.0000 | 0.0000 | 0.0062 | 1.0000 | 1.0000 |
